# Levels, trends and inequalities in mortality among 5–19-year-olds in Tanzania: Magu Health and Demographic Surveillance Study (1995–2022)

**DOI:** 10.7189/jogh.14.04124

**Published:** 2024-07-26

**Authors:** Sophia Kagoye, Eveline T Konje, Jim Todd, Charles Mangya, Mark Urassa, Abdoulaye Maïga, Milly Marston, Ties Boerma

**Affiliations:** 1 National Institute for Medical Research, Mwanza Research Centre, Mwanza, Tanzania; 2 Department of Epidemiology and Biostatistics, Catholic University of Health and Allied Sciences, Mwanza Tanzania; 3 Department of Population Health, London School of Hygiene and Tropical Medicine, London, UK; 4 Department of International Health, Johns Hopkins University Bloomberg School of Public Health, Baltimore, Maryland, USA; 5 Community Health Science, University of Manitoba, Winnipeg, Manitoba, Canada

## Abstract

**Background:**

For the past two decades, health priorities in Tanzania have focussed on children under-five, leaving behind the older children and adolescents (5–19 years). Understanding mortality patterns beyond 5 years is important in bridging a healthy gap between childhood to adulthood. We aimed to estimate mortality levels, trends, and inequalities among 5–19-year-olds using population data from the Magu Health and Demographic Surveillance Site (HDSS) in Tanzania and further compare the population level estimates with global estimates.

**Methods:**

Using data from the Magu HDSS from 1995 to 2022, from Kaplan Meir survival probabilities, we computed annual mortality probabilities for ages 5–9, 10–14 and 15–19 and determined the average annual rate of change in mortality by fitting the variance weighted least square regression on annual mortality probabilities. We compared 5–19 trends with younger children aged 1–4 years. We further disaggregated mortality by sex, area of residence and wealth tertiles, and we computed age-stratified risk ratios with respective 95% confidence intervals (CIs) using Cox proportional hazard model to determine inequalities. We further compared population-level estimates in all-cause mortality with global estimates from the United Nations Inter-agency Group for Child Mortality Estimation and the Global Burden of Disease study by computing the relative differences to the estimates.

**Results:**

Mortality declined steadily among the three age groups from 1995 to 2022, whereby the average annual rate of decline increased with age (2.2%, 2.7%, and 2.9% for 5–9-, 10–14-, and 15–19-year-old age groups, respectively). The pace of this decline was lower than that of younger children aged 1–4 years (4.8% decline). We observed significant mortality inequalities with boys, those residing in rural areas, and those from poorest wealth tertiles lagging behind. While Magu estimates were close to global estimates for the 5–9-year-old age group, we observed divergent results for adolescents (10–19 years), with Magu estimates lying between the global estimates.

**Conclusion:**

The pace of mortality decline was lower for the 5–19-year-old age group compared to younger children, with observable inequalities by socio-demographic characteristics. Determining the burden of disease across different strata is important in the development of evidence-based targeted interventions to address the mortality burden and inequalities in this age group, as it is an important transition period to adulthood.

Children and adolescents under 20 years of age account for 33% of the population globally and 52% of the population in sub-Saharan Africa [1], as well as more than 45% of the population in Tanzania [2]. Trends in mortality among children under five years are well studied [3–6]; however, the focus on mortality shifts to adulthood, ignoring the middle age group beyond five years, which is an important transition stage to adulthood [7]. Less is known about mortality among older children and adolescents; yet estimates for 2021 suggested that over 1 million deaths occurred at ages 5–19 years globally, including 700 000 in sub-Saharan Africa [8–10]. Therefore, reliable and timely data on levels, trends, and inequalities in mortality in this age group are important for comprehensive planning, programming, and monitoring of health and social services [11,12].

In the absence of well-functioning civil registration and vital statistics systems [13,14], household surveys are the primary source of data on mortality in low- and middle-income countries (LMICs). For Tanzania, national household surveys, primarily the Demographic and Health Surveys (DHS), are the main data source of global all-cause mortality estimates for the 5–19-year-old age group [10,15]. However, in the absence of empirical data, the assumptions behind the global estimates do not reflect the realities at a population level. Survey reports generally do not include mortality statistics for older children and adolescents because of large uncertainty due to smaller sample sizes.

In several countries in sub-Saharan Africa (including Tanzania), local populations are followed up using standardised procedures as part of prospective health and demographic surveillance systems (HDSSs) to collect vital event data on births, deaths and migration [14]. For example, the Magu HDSS is located in a rural area in northwestern Tanzania, and its population, socioeconomic, and health indicators are considered typical for the country as a whole [16]. As the Magu HDSS has been in operation since 1994, its longitudinal nature provides an opportunity for studying the long-term trends in mortality for the 5–19-year-old age groups during a period characterised by a strong focus on the survival of children under-five years, a major human immunodeficiency virus (HIV) epidemic, rapid population growth, and increasing education and economic growth [16,17] – factor which are unlikely to be captured in cross-sectional surveys.

In this study, we estimated long-term trends in all-cause mortality rate among children aged 5–9, 10–14 and 15–19 years during 1995–2022, using population data from Magu HDSS, and compared the changes in trends among older children and adolescents with that among younger children aged 1–4 years. We also examined the differences between the HDSS levels and trends and global estimates for Tanzania, and assessed inequalities within the Magu HDSS itself by disaggregating the mortality estimates by sex, place of residence, and wealth tertiles.

## METHODS

### Study setting

**Figure 1:**
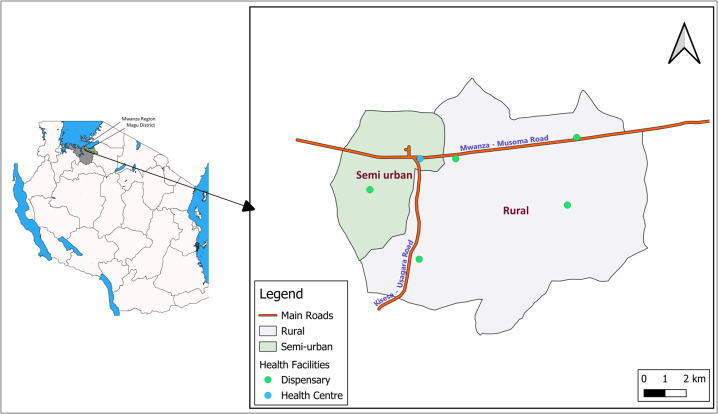
Map of Tanzania with the location of the Magu HDSS showing semi-urban and rural residence boundaries and location of health facilities.

The Magu HDSS is located in Kisesa ward, Magu district, Mwanza region, Tanzania. Kisesa ward is one of the 31 wards of the Magu district, while Magu is one of seven districts of the Mwanza region in northwestern Tanzania. The Magu HDSS is an open community cohort, which has been in operation in a contiguous area of seven villages since 1994 (administratively expanded to nine villages from 2018), located 20 km east of the city of Mwanza. Within the area, the villages closest to Mwanza city and that are part of or near the trading centre are considered as semi-urban, while all others are considered rural. The semi-urban and rural boundaries in Magu HDSS have not changed since 1994 and the distribution of the population in semi-urban/rural villages in Magu HDSS is 49% to 51% (**Figure 1**).

The population in the Magu HDSS has expanded from 19 347 in 1994 to 54 024 in 2022, with an annual population growth rate of 3.7% per year from 1994 to 2022. The population is and has remained young over time with 46–48% of it being under 15 years of age. The total fertility rate among women 15–49 years of age was 4.5 between 2018 and 2021, a decline from 6.3 in 1996. In our prior comparison of a range of demographic, socioeconomic, and health indicators, we showed that the Magu HDSS characteristics were very similar to Tanzania as a whole [16].

### Data collection

The data are collected by a team of nine local field workers who reside in the areas, with full-time supervision and regular monitoring visits from the National Institute for Medical Research. Each round of demographic surveillance was preceded by a training of field workers.

The Magu HDSS monitored birth, death, cause of death, and migration in the general population at intervals of approximately eight months since its conception in 1994, making a total of 40 HDSS rounds until 2022. All residents of households within the HDSS area are registered through home visits using a demographic household card with their names, age, sex, and relation to the household head. An HDSS number is allocated to each listed individual based on administrative subdivisions in the HDSS area, i.e. village, sub-village, ten household leaders, household number, and line number of a person in the household card. The data collection process was paper-based from 1994 to 2012 (HDSS round 26), after which the update of the HDSS data switched to an electronic format by the use of personal digital assistants (PDAs). Information is currently collected using tablets, with data entry and management handled through the Census and Survey Processing System (CSPro), version 8 (USA Census Bureau, USA) [18]. Regular data quality tables were run to detect any problems in the field work. Data quality issues that emerged during merging with previous rounds were addressed through consultation with the field team, after which the full data set was finally edited and cleaned for analysis. The process of reconciling data from multiple rounds became more efficient from 2012 onward with the introduction of PDAs and (eventually) tablets. Detailed information on HDSS data collection methods are given elsewhere [17].

### Measurements

To calculate mortality, we created a residency episodes file using data form each round, defining it for each period an individual aged 5–19 years spent in the Magu HDSS. We then used this file to calculate the total person-years of observation in this analysis. The Magu HDSS defines residency as living for three months or more in the study area [17]. All residency episodes started at birth, in migration, or the date first seen in Magu HDSS (including at the beginning of the study); they finished at death, outmigration, or the date last seen as a lost-to-follow-up or the right censoring date (31 December 2022). A new person (in-migrant) was listed as a household member if the household respondent had stayed or was intending to stay in the household for at least three months. Those who left a household in the Magu HDSS and were not expected to return to that household were considered out-migrants from the household, but maintained their line number, allowing returning household members to be relisted according to their original HDSS numbers. Dates of in- and out-migration were recorded for older children and adolescents who moved out of households and changed residences.

We measured household wealth tertiles as a proxy indicator using information on the household asset indicators that include construction materials of the main dwelling; type of toilet facilities; sources of water and energy; ownership of modern assets; and livestock (Table S1 in the **Online Supplementary Document**). Our rationale of using household asset indicators as a measure of socioeconomic status over ‘direct’ measures such as income, expenditure, and financial assets (e.g. savings and pensions) was their reliability over time and cost-effectiveness, as the information is relatively easy and inexpensive to collect. Additionally, household assets provide a better proxy for a household’s long-run wealth compared to information on income or expenditures; this is due to seasonal variability in earnings, income from potentially multiple and diverse informal activities, high rates of self-employment, possible recall bias, and misreporting [19]. Information on household assets was updated three more times from the baseline census as part of demographic rounds in 2004/05, 2018 and 2022. We used principal component analysis (PCA) to construct a wealth score, which we divided into three equal parts as wealth tertiles [19,20].

### Statistical analysis

Using time-to-event analysis with age as a time scale, we obtained the total number of deaths and the total person years of observation annually from full periods of observation (January 1995 to December 2022) which were grouped into four-year periods. Entry to risk began at birth, in-migration, or study start date. Exit from risk was at the earliest of study end date (December 2022), out-migration, or death. If an out-migration is followed by an in-migration, the period between the out- and in-migration was excluded from the risk period to avoid survivor bias.

We calculated crude mortality rates (CMR) for years 1995 to 2022 by dividing the total number of deaths with total person years of observation at that year, per the following equation:

*CMR_j_* = *d_j_ /n_j_*

Here, *n_j_* denotes the total person-years of older children and adolescents aged 5–9, 10–14, and 15–19 years at year *j* = 1995–2022, respectively, while *d_j_* denotes the corresponding number of deaths.

Further, we computed age-specific mortality probabilities or risks and their 95% confidence intervals (CIs) from Kaplan-Meier survival probabilities at 5-year age groups, i.e. the probability of a 5-year-old dying before reaching the 10th birthday (*_5_q_5_*), the probability of a 10-year-old dying before reaching the 15th birthday (*_5_q_10_*), and a probability of a 15-year-old dying before reaching the 20th birthday (*_5_q_15_*).

The Magu HDSS present a census, and not a sample. However, we computed 95% CIs for all mortality statistics using Poisson regression models and presented these in the **Online Supplementary Document** to give an indication of uncertainty related to the number of observations and assess the differences between subgroups.

To determine the average annual rate of change (AARC) in age-specific mortality probabilities from 1995 to 2022, we fitted a variance weighted least square regression to annual mortality probabilities. We presented the AARC with respective 95% CIs. We also computed the AARC for child mortality trends (1–4 years) to compare them with trends among older children and adolescents (5–19 years). We selected the 1–4-year-old age group because it appeared that infant mortality (particularly neonatal mortality) was underestimated, as has been found in other HDSS assessments [21].

We analysed the data in the subgroups of sex, geographical location (semi-urban/rural residence), and household wealth tertiles. We used the semi-urban to rural grouping of villages which had shown to be a major factor in the HIV epidemic [22]. For socioeconomic status, we were able to link 74.8% of individual observations with household wealth data. Based on the asset score, we classified households into wealth tertiles, i.e. the poorest, middle, and richest 33%, to obtain adequate numbers of observations in each category.

We examined sociodemographic inequalities of all-cause mortality risk among older children and adolescents using an age stratified Cox proportional hazards model, disaggregated by sex, area of residence, and wealth tertiles, presenting the measure of effect using hazards ratio with corresponding 95% CIs. Here, a *P*-value of <0.05 denoted statistical significance.

We compared the population data findings from the Magu HDSS with the global estimates for Tanzania by computing relative differences for 4-year averages from 1995 onward for the mortality of 1–4-, 5–9-, 10–14-, and 15–19-year-old-age groups.

The United Nations Interagency Group on Child Mortality estimates (UN IGME) uses nationally representative data to estimate child mortality; in countries with no reliable vial registration data specifically, it uses household deaths from population census and household surveys (DHS). Mortality rates among children aged 1–4 years and older children and younger adolescents (5–14 years) are derived from full birth histories, while mortality among older adolescents (15–19 years) are derived from sibling survival histories. Recalculation are then done from data inputs and adjustments, after which a statistical model is fit to these data to generate a smooth trend curve and further extrapolate the model to a target year [10].

The Global Burden of Disease (GBD) study utilises available data sources from household surveys, population censuses, health and demographic surveillance, and epidemiological studies to estimate under-five and adult mortality rates. There are no specific underlying data sources for the estimation of mortality among older children and adolescents; mortality estimates for this age group are obtained using model life tables with two entry parameters: the under-five mortality rate and adult mortality to obtain age-specific mortality probabilities [23].

We defined the relative difference as the difference between the population data estimate and each global estimate (UN IGME or GBD) divided by the Magu HDSS estimate [24] so as to put the population data estimates in the context of other modelled estimates and to test the external validity of our population data.

We analysed all data in Stata, version 18.0 (StataCorp LLC, College Station, Texas, USA).

## RESULTS

### Mortality levels and trends (1995–2022)

We observed 635 deaths and 328 078.8 person years among older children and adolescents (5–19 years), with 308 deaths and 131 507.5 person years among older children 5-9 years, 148 deaths and 111 432.5 person years among younger adolescents (10–14 years), and 179 deaths and 85 139.8 person years among older adolescents (15–19 years) (Table S2 in the **Online Supplementary Document**).

**Figure 2:**
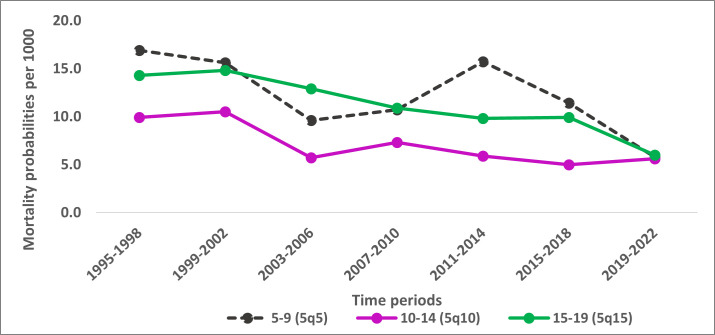
Mortality per 1000 among older children (5–9 years), younger adolescents (10–14 years), and older adolescents (15–19 years) in the Magu HDSS by 4-year time periods (1995–2022).

Mortality was the lowest in the 10–14-year-old group compared to the 5–9- and 15–19-year-olds, although the gap in mortality between the three age groups was small during the last period. Mortality was 5.8, 5.6, and 6.0 deaths per 1000 during the final period (2019–22) for older children aged 5–9 years, adolescents aged 10–14, and 15–19 years, respectively (**Figure 2**).

Mortality among children aged 5–9 years (*_5_q_5_*) fluctuated over time, with a rapid decline in the first decade (from 16.9 to 9.6 deaths per 1000). However, we then observed a reversal in the trend, peaking in the 2011–14 period to 15.7, followed by a second rapid decline to 5.8 deaths per 1000 in the final, 2019–22 period (**Figure 2**). Mortality among adolescents (10–14 and 15–19 years) showed two periods with higher mortality in the earlier years, followed by a decline from 2003 in both age groups. However, we observed a period with no steady improvement in adolescents’ survival from 2011 to 2019–22 among 10–14-year-olds (ranging between 5.9 to 5.0 deaths per 1000) and from 2007 to 2018 among 15–19-year-olds (ranging between 10.9 to 9.8 deaths per 1000) (**Figure 2**).

There was a strong, sustained mortality decline in all age groups (**Figure 2**). The annual rate of mortality declines since 1995 to 2022 among older children and adolescents increased with age, with older children (5–9 years) having the lowest annual rate of decline of 2.0%, followed by younger adolescents (10–14 years) with a decline of 2.7% and older adolescents (15–19 years) with the highest rate of decline of 2.9% per year. The pace of mortality decline in all three age groups (5–9, 10–14, and 15–19 years) was much lower compared to the 1–4-year-old age group, where mortality declined at 4.8% per year. (Table S9 in the **Online Supplementary Document**).

### Mortality inequalities

**Figure 3:**
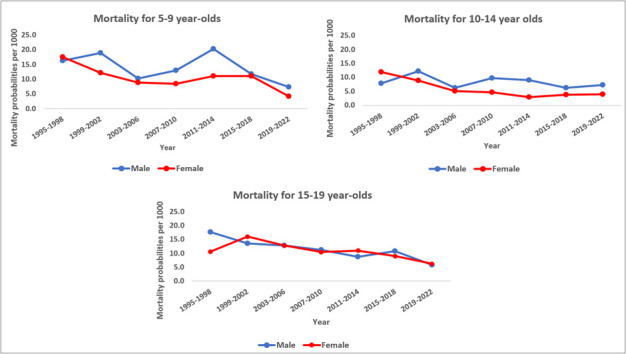
Mortality per 1000 among older children and adolescents aged 5–9, 10–14, and 15–19 years in the Magu HDSS by sex and 4-year time periods (1995–2022).

**Figure 4:**
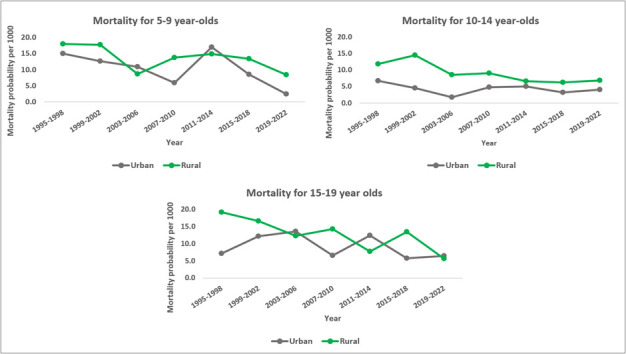
Mortality probabilities per 1000 among older children and adolescents aged 5–9, 10–14, and 15–19 years in the Magu HDSS by area of residence and 4-year time periods (1995–2022).

**Figure 5:**
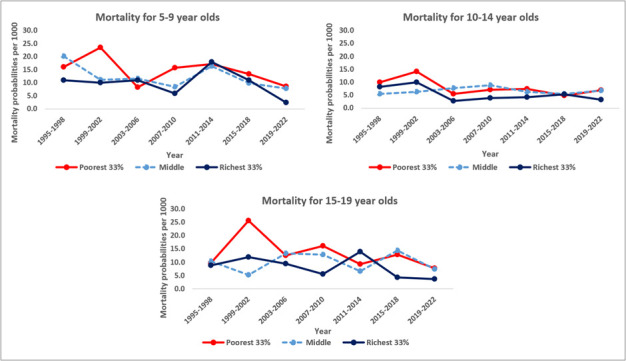
Mortality probabilities per 1000 among older children and adolescents aged 5–9, 10–14, and 15–19 years in the Magu HDSS by wealth tertiles and 4-year time periods (1995–2022).

Mortality declined for both sexes but females had a higher annual rate of decline in mortality compared to males among 5–14-year-olds (children aged 5–9 years: 2.6% vs 2.0%, younger adolescents aged 10–14 years: 6.4% vs 1.3%). Older adolescents (15–19 years) showed a similar rate of mortality decline among both males and females at 2.5% decline every year. This is in contrast to younger children aged 1–4 years, where mortality declined faster among males compared to females (5.1% vs 4.3%) (**Figure 3**, **Figure 4**, **Figure 5**; Tables S3–5 and S9 in the **Online Supplementary Document**).

Mortality risk was higher among males compared to females for 5–9- and 10–14-year-olds. After adjusting for other factors, mortality risk ratio was 44% higher and 51% higher among males aged 5–9 years (adjusted hazard ratio (aHR) = 1.44; 95% CI = 1.13–1.82) and 10–14 years (aHR = 1.51; 95% CI = 1.06–2.14), respectively, compared to females. There were no significant differences in mortality by sex among older adolescents aged 15–19 years (**Table 1**).

**Table 1:** Socio-demographic determinants of all-cause mortality risk among older children and adolescents (5–19 y) (age stratified Cox proportional hazard model), 1995–2022

				5–9 years				10–14 years				15–19 years			
	**Crude HR (95% CI)**	***P*-value**	aHR (95% CI)*		***P*-value**	**Crude HR (95% CI)**	***P*-value**		aHR (95% CI)*	***P*-value**	**Crude HR (95% CI)**		***P*-value**	aHR (95% CI)*	***P*-value**
**Time periods**															
1995–98	2.92 (1.86–4.58)	<0.001	2.60 (1.59–4.25)		<0.001	1.71 (0.96–3.03)	0.067		1.27 (0.64–2.52)	0.433	2.39 (1.35–4.23)		0.003	1.54 (0.78–3.04)	0.207
1999–2002	2.68 (1.71–4.19)	<0.001	2.54 (1.59–4.04)		<0.001	1.80 (1.04–3.10)	0.035		1.77 (1.00–3.13)	0.050	2.49 (1.43–4.35)		0.001	2.36 (1.33–4.19)	0.003
2003–06	1.64 (1.01–2.66)	0.047	1.58 (0.97–2.57)		0.054	1.01 (0.55–1.87)	0.967		0.97 (0.52–1.83)	0.987	2.17 (1.25–3.77)		0.006	1.89 (1.07–3.34)	0.026
2007–10	1.84 (1.16–2.92)	0.010	1.74 (1.08–2.79)		0.018	1.25 (0.72–2.19)	0.429		1.13 (0.63–2.04)	0.635	1.75 (1.00–3.07)		0.050	1.78 (1.01–3.12)	0.043
2011–14	2.72 (1.79–4.14)	<0.001	2.75 (1.80–4.19)		<0.001	1.03 (0.58–1.83)	0.923		1.06 (0.59– 1.90)	0.811	1.59 (0.90–2.80)		0.109	1.57 (0.89–2.78)	0.165
2015–18	1.96 (1.26–3.05)	0.003	1.88 (1.20–2.94)		0.005	0.86 (0.47–1.55)	0.606		0.89 (0.49–1.61)	0.717	1.56 (0.89–2.74)		0.118	1.57 (0.90–2.75)	0.113
2019–22	ref		ref			ref			ref		ref			ref	
**Sex**															
Male	1.40 (1.12–1.76)	0.003	1.44 (1.13–1.82)		0.003	1.54 (1.11–2.13)	0.010		1.51 (1.06–2.14)	0.021	1.09 (0.81–1.46)		0.573	1.03 (0.75–1.40)	0.792
Female	ref		ref			ref			ref		ref			ref	
**Area of residence**															
Semi-urban	ref		ref			ref			ref		ref			ref	
Rural	1.37 (1.08–1.73)	0.009	1.09 (0.82–1.45)		0.206	2.07 (1.44–1.98)	<0.001		1.98 (1.27–3.09)	0.001	1.35 (0.99–1.83)		0.052	0.97 (0.67–1.39)	0.844
**Wealth tertiles**															
Poor	1.67 (1.23–2.25)	0.009	1.48 (1.05–2.10)		0.020	1.72 (1.08–2.72)	0.045		1.13 (0.67–1.92)	0.852	1.78 (1.19–2.67)		0.004	1.74 (1.09–2.78)	0.019
Middle	1.24 (0.89–1.72)	0.143	1.15 (0.82–1.62)		0.477	1.77 (1.07–2.73)	0.047		1.35 (0.82–2.23)	0.646	1.56 (1.02–2.36)		0.026	1.52 (0.98–2.35)	0.285
Rich	ref		ref			ref			ref		ref			ref	

aHR – adjusted hazard ratio, CI – confidence interval, ref – reference

*Adjusted for time periods– sex– area of residence and household wealth tertiles.

Mortality within the HDSS declined quicker in rural than in semi-urban villages for older children (5–9 years) and younger adolescents (10–14 years) (rural vs semi-urban children (5–9 years): 1.8% vs 1.2%, rural vs semi-urban younger adolescents (10-14 years): 2.8% vs 1.7%). Mortality decline among older adolescents (15–19 years) was similar in both areas (rural: 3.2%, semi-urban: 3.1%). Among younger children (1–4 years), there was a faster mortality decline in semi-urban villages (5.6% per year) compared to rural villages (4.1% per year) (Table S9 in the **Online Supplementary Document**).

Mortality was higher in the rural than in the semi-urban villages in all three age groups. This remained statistically significant for only younger adolescents after adjusting for household wealth and other factors. The mortality risk ratio among younger adolescents (10–14 years) residing in rural areas increased by 98% compared to their counterparts in urban areas (aHR = 1.98; 95% CI = 1.27–3.09). Although not statistically significant, we found lower mortality risk ratio in rural areas compared to their counterparts among older adolescents (15–19 years) (aHR = 0.97; 95% CI = 0.67–1.39).

The mortality risks by household wealth tertiles differed significantly among children aged 5–9 years and older adolescents aged 15–19 years (**Table 1**). The risk was higher among the poorest followed by the middle wealth tertiles, as compared to the richest wealth tertile. For 5–9-year-olds, those from poorest and middle tertiles had a 48% (aHR = 1.48; 95% CI = 1.05–2.10) and 15% (aHR = 1.15; 95% CI = 0.82–1.62) higher mortality risk compared to those from the richest wealth tertile, respectively. For adolescents (15–19-year-olds), those in the poorest wealth tertile had 74% higher risk of dying (aHR = 1.74; 95% CI = 1.09–2.78), while those from the middle tertile had a 52% higher risk of dying compared to those in the richest wealth tertile (aHR = 1.52; 95% CI = 0.98–2.35).

### Comparison with global estimates

The mean absolute difference in four-year mortality risks for the three age groups combined (5–19 years) was 2.1 per 1000 between the HDSS and UN estimates, 2.7 per 1000 between the HDSS and the GBD study, and 3.4 per 1000 between the two global estimates.

**Figure 6:**
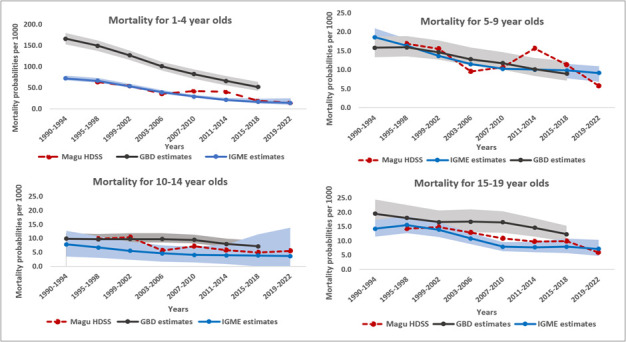
All-cause mortality estimates for 1–4, 5–9-, 10–14-, and 15–19-year-olds comparing global estimates from the UN IGME [25], GBD [26], and population data from the Magu HDSS.

At 5–9-years, the Magu HDSS mortality risks were comparable to those estimated by the UN IGME, with relative differences ranging from 3–20%. For the GBD study, the Magu mortality risks were comparable in 1995–98, 1999–2002, and 2007–10, with relative differences ranging from 5–10%. We observed differences in the 2003–06 period, with lower mortality rates in Magu (33.8% difference with GBD estimates). We also found major differences between th eMagu HDSS data and global estimates in the 2011–14 period, with higher estimated mortality in the Magu HDSS by 35.8% and 35.2% compared to UNIGME and GBD study estimates, respectively (**Figure 6** and Table S10 in the **Online Supplementary Document**).

The two global estimates for mortality among younger adolescents (10–14 years) diverge widely, with GBD study estimates being higher than the UN ones. The Magu HDSS results showed an intermediate pattern, closer to the GBD study estimates during the earlier periods (relative differences of 1.9% and 6.5% in 1995–98 and 1999–2002 periods) and closer to the UN IGME trend in more recent years (relative difference 21.5% in 2015–18 period compared to 44% with the GBD study).

For the 15–19-year-olds, the HDSS trends were similar to the UN estimates (relative difference ranging from 6% to 26.8%), while the GBD study estimates for Tanzania were higher during all periods, with relative differences of up to 51.3%.

We also compared 1–4 mortality estimates from the Magu HDSS with global estimates. Here, the Magu HDSS estimates were comparable to the UN IGME ones across the time periods, with relative differences ranging from 1.3% to 11%. However, we observed differences in 2007–10 and 2011–14 periods, with relative differences of 31.9% and 46.9%, respectively. We otherwise saw major differences with the GBD study estimates, with the Magu HDSS estimating lower rates by at least 65.4%.

## DISCUSSION

The Magu HDSS analyses provide insights into mortality levels, trends, and inequalities among older children and adolescents (aged 5–19 years) during the 1995–2022 period. This study is the first of its kind in Tanzania and one of the few long-term assessments at a population level for this age group in sub-Saharan Africa [27–31].

Overall, the risk of dying was lowest among younger adolescents 10–14 years and highest among older children aged 5–9 years. This is similar to what was observed in other LMICs [29,32] and in contrast to high-income countries, where the risk of dying is lowest at ages 5–9 years [32]. The higher mortality burden at ages 5–9 in our study suggests that there is likely a continuing mortality risk from infectious childhood diseases. According to global estimates, infectious diseases (including malaria, diarrhoeal diseases, and HIV/AIDS) account for more than 50% of deaths at ages 5–9 in Tanzania [33]. Further work analysing the cause of death patterns in this age group within the HDSS is ongoing.

We observed a steady decline in mortality over time across the three age groups, although reversals of the downward mortality trend occurred in a few four-year periods. Children aged 5–9 years had a slower annual rate of decline in mortality (2.2%) compared to adolescents (2.7% and 2.9% decline for 10–14- and 15–19-year-olds, respectively). This is remarkable, given that there has been little specific health programme focus on this age group in Tanzania during the last two decades. However, the pace of mortality decline in this age group was about half of that among children 1–4 years (4.8% decline per year), similar to what was observed in other studies in LMICs [32]. Early childhood mortality (1–4 years) has been a major programmatic focus over time, which influenced a major mortality decline in this age group [34,35]. We did not find evidence of impact of the HIV epidemic on 5–19 years mortality, which had its greatest impact on adult mortality in the population around 2000–03 [18]. A refocus of policies and interventions on the age groups beyond five years is necessary to expedite reductions in mortality and improve quality of life in this age group, which encompasses a critical transition period to adulthood.

The Magu HDSS also allowed us to assess the inequalities within a local population in adjacent villages. We found large male excess mortality at 5–9 and 10–14 years: 1.44 and 1.51, respectively. Male excess mortality was much higher than that estimated by the UN-IGME for Tanzania, where the average male-female ratios were 1.04, 1.2, and 1.1 for the 5–9-, 10–14-, and 15–19-year-olds, respectively. This was higher compared to what was observed in other studies [36]. Additionally, mortality decline was slower among males compared to females across all age groups, similar to what has been observed elsewhere [27,36,37]. The potential reason for the observed sex differences in mortality is likely the differences in exposure to risk factors between males and females, with males being at a higher risk for injuries compared to females, which is one of the leading causes of death in this age group [29,31]. Further research on the underlying differences in cause-specific mortality by sex could help address sex-specific inequalities in this age group.

A second inequality concerned the place of residence. Our findings show that older children and younger adolescents (5–9 and 10–14 years, respectively) in semi-urban villages experienced much lower mortality risk than those in rural households. However, after controlling for wealth, this gap only remained statistically significant at the 10–14-year-old age group, where mortality was nearly two times higher among rural children within this small area of the HDSS. One possible explanation is the poor access to health services among those residing in rural areas, especially due to the distribution of lower-level facilities in the rural areas compared to semi-urban area, leading to the observed lagging.

In terms of wealth, individuals from the poorest wealth tertiles had a 70–80% higher mortality risk than those in the richest tertile. Such patterns of inequality have been reported by other studies [29], highlighting the need to focus health programmes on the poorest populations. Differences in place of residence could only explain a small part of these differences in mortality risk between the poorest and richest households.

Lastly, we compared our results with global estimates for Tanzania. As noted elsewhere, there is evidence that the Magu HDSS can be considered representative of the Tanzanian population, given the close correspondence for a wide range of population, socioeconomic, and health indicators over the past two decades [16]. Therefore, we expected our mortality estimates to be close to the global estimates for Tanzania. This was indeed the case for children 5–9 years, where there was little difference with the UN-IGME and the GBD study estimates. At ages 10–14 and 15–19 years, however, we observed major differences between the two global estimates; in both cases, with the Magu HDSS results falling in between in both cases. The discrepancies are likely related to the different empirical data and estimation methods used in each global estimate.

In general, our findings suggest that the global trend estimates of mortality 5–19 years are a useful source for Tanzania, though the wide difference between the global sources themselves needs to be addressed. It was surprising, however, that the differences between the two global estimates for Tanzania were larger than the differences between each global estimate and the results from the Magu HDSS. More and better national and subnational data on mortality 5–19 years are needed for tracking mortality in the future.

One of the limitations of our study is the relatively small number of child and adolescent deaths spread out over a long period, resulting in considerable mortality fluctuations. We partially reduced this bias by considering four-year periods and CIs, as well as by focussing on long-term trends. As in any death registration system, we may have missed deaths in the relevant age groups; however, the Magu HDSS has been characterised by an intensive and well-established system of follow up [17]. The consistency of the mortality results with other sources and over time suggests that omission was not a major issue. Furthermore, while migration may affect the denominators, our strict inclusion criteria to compute person years of observation have minimised this risk. Finally, our comparison with global estimates can only be tentative, as the Magu HDSS is a local study, even though it appears to be quite representative for Tanzania [16].

## CONCLUSION

Our study provides evidence on long-term trends and inequalities in child and adolescent mortality based on 28 years of demographic surveillance in a rural population in northwest Tanzania. Despite little programmatic focus on the older children and adolescent during 1995–2022, mortality risks in the 5–9-, 10–14-, and 15–19-year-old age groups declined to 5–6 per 1000 population. This decline was, however, was at an average annual rate of reduction that was only half of what was observed at ages 1–4 years. Improvements in survival occurred in all subgroups, but boys, children in rural villages, and children in the poorest households had considerably higher mortality than girls (5–14 years), children in semi-urban villages, and children in in the richest households, respectively. The trend data suggest that a greater focus on health and survival of children beyond age five years can accelerate the mortality decline, especially when focussed on the more disadvantaged children and adolescents. We also showed empirical data are an important input to enhance global estimation of mortality among older children and adolescents. Further investments in measurement and monitoring mortality and other health indicators in this age group are critical for successful programs.

## Additional material


Online supplementary Document
