# Supplementary material for: Levels, trends and inequalities in mortality among 5–19-year-olds in Tanzania: Magu Health and Demographic Surveillance Study (1995–2022)
Source: J Glob Health. 2024 Jul 26;14:04124. doi: 10.7189/jogh.14.04124 (PMC11271169; doi:10.7189/jogh.14.04124)
Supplement: Online supplementary Document [file jogh-14-04124-s001.pdf]

## Supplementary material

**Table S1: Household asset indicators used for creating asset-based wealth index in Magu HDSS (2004/05, 2018 and 2022)**

| | Indicator | Examples |
|---|---|---|
| 1 | Construction materials of the main dwelling | |
| | Type of wall material | Brick, Cement, other modern, covered adobe Traditional mud |
| | Type of roof material | Rustic, sod, cardboard, tiles, wood/timber, corrugated iron sheets, metal |
| | Type of floor material | Mud/Dirt/earth/sand/animal dung, cement, carpet, other modern, tiles, marble |
| | Number of bedrooms | 1 bedroom, 2 bedrooms, 3 bedrooms, 4 bedrooms, ≥5 bedrooms |
| 2 | Type of toilet facility | No toilet facility, bucket, traditional pit latrine (own/shared), ventilated improved pit latrine (own/shared), modern toilet flush somewhere else/trench/pit, modern toilet flush to sewer/septic tank |
| 3 | Source of water | Open source/unprotected, well/spring(protected), tap/piped water |
| 4 | Sources of energy | |
| | Cooking energy | Firewood, agricultural crop, residue/saw dust/waste, coal/charcoal/briquettes, electricity/gas, LPG, Kerosene/paraffin |
| | Lighting energy | Candles/charcoal/biogas, Kerosene/paraffin, battery, solar, electricity/gas |

**Table S2: Number of deaths and mortality probabilities per 1000 among older children and adolescents (5-9, 10-14 and 15-19 years) in Magu HDSS by 4-year time periods (1995-2022)**

| Time period | 5-9 years | | | 10-14 years | | | 15-19 years | | |
|---|---|---|---|---|---|---|---|---|---|
| | Deaths | Total person years | Mortality probability ($_5q_5$) | Deaths | Total person years | Mortality probability ($_5q_{10}$) | Deaths | Total person years | Mortality probability ($_5q_{15}$) |
| 1995-1998 | 44 | 12741.9 | 16.9 (10.5, 27.2) | 20 | 10277.1 | 9.9 (5.3, 18.4) | 24 | 8216.5 | 14.3 (8.5, 24.1) |
| 1999-2002 | 45 | 14238.5 | 15.6 (9.5, 25.6) | 24 | 11768.6 | 10.5 (5.7, 19.2) | 27 | 8868.4 | 14.8 (8.9, 24.6) |
| 2003-2006 | 32 | 16570.1 | 9.6 (5.1, 18.1) | 16 | 13841.5 | 5.7 (2.5, 13.0) | 28 | 10549.2 | 12.9 (7.5, 22.2) |
| 2007-2010 | 40 | 18432.3 | 10.7 (5.9, 19.5) | 22 | 15419.4 | 7.3 (3.5, 15.0) | 26 | 12154.0 | 10.9 (6.0, 19.7) |
| 2011-2014 | 66 | 20504.5 | 15.7 (9.6, 25.8) | 20 | 17101.7 | 5.9 (2.6, 13.3) | 25 | 12856.7 | 9.8 (5.2, 18.3) |
| 2015-2018 | 48 | 20916.8 | 11.4 (6.4, 20.4) | 18 | 18506.8 | 5.0 (2.1, 12.0) | 26 | 13616.8 | 9.9 (5.3, 18.5) |
| 2019-2022 | 33 | 28103.4 | 5.8 (2.6, 13.1) | 28 | 24517.4 | 5.6 (2.5, 12.8) | 23 | 18877.2 | 6.0 (2.7, 13.4) |

**Table S3: Mortality probabilities per 1000 among older children and adolescents (5-9, 10-14 and 15-19 years) in Magu HDSS by sex and 4-year time periods (1995-2022)**

| Time period | 5-9 | | 10-14 | | 15-19 | |
|---|---|---|---|---|---|---|
| | Male | Female | Male | Female | Male | Female |
| 1995-1998 | 16.3 (10.1, 26.5) | 17.5 (11.0, 28.0) | 7.9 (3.9, 15.8) | 11.9 (6.7, 20.9) | 17.6 (11.1, 28.1) | 10.6 (5.8, 19.4) |
| 1999-2002 | 18.9 (12.0, 29.7) | 12.2 (6.9, 21.4) | 12.2 (6.9, 21.4) | 8.8 (4.5, 17.0) | 13.6 (8.0, 23.1) | 16.0 (9.8, 26.1) |
| 2003-2006 | 10.3 (5.6, 18.9) | 8.9 (4.6, 17.1) | 6.3 (2.9, 13.7) | 5.1 (2.1, 12.1) | 12.9 (7.5, 22.3) | 12.8 (7.4, 22.2) |
| 2007-2010 | 13.0 (7.6, 22.4) | 8.4 (4.3, 16.5) | 9.8 (5.3, 18.4) | 4.6 (1.8, 11.5) | 11.2 (6.2, 20.1) | 10.5 (5.7, 19.2) |
| 2011-2014 | 20.3 (13.1, 31.4) | 11.0 (6.1, 19.9) | 9.0 (4.7, 17.4) | 2.9 (0.9, 9.2) | 8.8 (4.5, 17.0) | 11.0 (6.1, 19.9) |
| 2015-2018 | 11.8 (6.7, 20.9) | 11.0 (6.1, 19.8) | 6.2 (2.8, 13.6) | 3.8 (1.4, 10.4) | 10.8 (6.0, 19.7) | 9.0 (4.7, 17.3) |
| 2019-2022 | 7.4 (3.6, 15.3) | 4.2 (1.6, 10.9) | 7.2 (3.5, 15.0) | 4.0 (1.5, 10.7) | 5.9 (2.7, 13.3) | 6.2 (2.8, 13.6) |

**Table S4: Mortality probabilities per 1000 among older children and adolescents (5-9, 10-14 and 15-19 years) in Magu HDSS by area of residence and 4-year time periods (1995-2022)**

| Time period | 5-9 | | 10-14 | | 15-19 | |
|---|---|---|---|---|---|---|
| | Semi-urban | Rural | Semi-urban | Rural | Semi-urban | Rural |
| 1995-1998 | 15.0 (9.1, 24.9) | 18.0 (11.3, 28.6) | 6.7 (3.2, 14.3) | 11.9 (6.7, 21.0) | 7.2 (3.5, 15.0) | 19.2 (12.3, 30.1) |
| 1999-2002 | 12.6 (7.3, 21.9) | 17.7 (11.1, 28.2) | 4.5 (1.8, 11.4) | 14.5 (8.7, 24.3) | 12.2 (7.0, 21.4) | 16.6 (10.3, 26.9) |
| 2003-2006 | 10.9 (6.0, 19.7) | 8.7 (4.5, 17.0) | 1.8 (0.4, 7.8) | 8.5 (4.3, 16.7) | 13.6 (8.0, 23.1) | 12.3 (7.0, 21.5) |
| 2007-2010 | 6.0 (2.7, 13.3) | 13.8 (8.2, 23.4) | 4.8 (2.0, 11.8) | 9.0 (4.7, 17.3) | 6.6 (3.1, 14.2) | 14.3 (8.5, 24.0) |
| 2011-2014 | 17.0 (10.6, 27.4) | 14.9 (9.0, 24.7) | 5.0 (2.1, 12.0) | 6.6 (3.1, 14.2) | 12.4 (7.1, 21.6) | 7.7 (3.8, 15.6) |
| 2015-2018 | 8.6 (4.4, 16.7) | 13.4 (7.9, 22.9) | 3.2 (1.1, 9.6) | 6.3 (2.9, 13.7) | 5.7 (2.5, 13.0) | 13.5 (7.9, 23.0) |
| 2019-2022 | 2.5 (0.7, 8.6) | 8.4 (4.2, 16.5) | 4.1 (1.5, 10.7) | 6.9 (3.3, 14.5) | 6.5 (3.0, 14.1) | 5.6 (2.5, 12.8) |

**Table S5: Mortality probabilities per 1000 among older children and adolescents (5-9, 10-14 and 15-19 years) in Magu HDSS by wealth tertiles and 4-year time periods (1995-2022)**

| Time period | 5-9 | | | 10-14 | | | 15-19 | | |
|---|---|---|---|---|---|---|---|---|---|
| | Poorest 33% | Middle | Richest 33% | Poorest 33% | Middle | Richest 33% | Poorest 33% | Middle | Richest 33% |
| 1995-1998 | 16.1 (9.9, 26.3) | 20.1 (13.0, 20.1) | 11.0 (6.1, 19.9) | 9.9 (5.3, 18.5) | 5.5 (2.4, 12.7) | 8.2 (4.1, 16.2) | 9.7 (5.2, 18.2) | 10.4 (5.7, 19.1) | 8.8 (4.5, 17.0) |
| 1999-2002 | 23.5 (15.7, 35.2) | 11.1 (6.2, 20.0) | 10.1 (5.5, 18.7) | 14.1 (8.3, 23.7) | 6.3 (2.9, 13.7) | 10.0 (5.4, 18.6) | 25.6 (17.4, 37.7) | 5.3 (2.3, 12.5) | 11.9 (6.7, 21.0) |
| 2003-2006 | 8.3 (4.2, 16.4) | 11.6 (6.5, 20.6) | 10.9 (6.0, 19.8) | 5.5 (2.4, 12.6) | 7.7 (3.8, 15.6) | 2.8 (0.9, 9.1) | 12.5 (7.2, 21.8) | 13.4 (7.8, 22.9) | 9.5 (5.0, 17.9) |
| 2007-2010 | 15.8 (9.6, 15.8) | 8.4 (4.2, 16.5) | 5.9 (2.6, 13.2) | 7.1 (3.4, 14.8) | 8.8 (4.6, 17.1) | 3.8 (1.4, 10.5) | 16.1 (9.9, 26.2) | 12.8 (7.4, 22.1) | 5.5 (2.4, 12.7) |
| 2011-2014 | 17.1 (10.6, 27.4) | 16.3 (10.1, 26.5) | 18.0 (11.3, 28.5) | 7.4 (3.6, 15.2) | 6.3 (2.9, 13.7) | 4.2 (1.6, 11.0) | 9.3 (4.9, 17.7) | 6.7 (3.2, 14.3) | 14.0 (8.3, 23.6) |
| 2015-2018 | 13.4 (7.8, 22.8) | 9.8 (5.3, 18.4) | 11.0 (6.1, 19.9) | 4.9 (2.0, 11.9) | 5.4 (2.3, 12.6) | 5.3 (2.2, 12.4) | 12.8 (7.4, 22.2) | 14.4 (8.6, 24.1) | 4.4 (1.7, 11.2) |
| 2019-2022 | 8.6 (4.4, 16.8) | 7.8 (3.8, 15.7) | 2.4 (0.7, 8.5) | 6.9 (3.2, 14.5) | 6.8 (3.2, 14.4) | 3.3 (1.1, 9.8) | 7.7 (3.8, 15.6) | 7.4 (3.6, 15.2) | 3.7 (1.3, 10.3) |

**Table S6: Number of deaths and total person years among older children and adolescents (5-9, 10-14 and 15-19 years) in Magu HDSS by sex and 4-year time periods (1995-2022)**

| Time period | 5-9 | | | | 10-14 | | | | 15-19 | | | |
|---|---|---|---|---|---|---|---|---|---|---|---|---|
| | Male | | Female | | Male | | Female | | Male | | Female | |
| | Deaths | Total person years | Deaths | Total person years | Deaths | Total person years | Deaths | Total person years | Deaths | Total person years | Deaths | Total person years |
| 1995-1998 | 21 | 6406.1 | 23 | 6335.8 | 8 | 5144.7 | 12 | 5132.4 | 16 | 4423.3 | 8 | 3793.2 |
| 1999-2002 | 28 | 7235.0 | 17 | 7003.4 | 14 | 5926.3 | 10 | 5842.3 | 13 | 4724.4 | 14 | 4144.0 |
| 2003-2006 | 17 | 8244.8 | 15 | 8325.3 | 9 | 7080.4 | 7 | 6761.1 | 15 | 5544.9 | 13 | 5004.4 |
| 2007-2010 | 24 | 8860.0 | 16 | 9572.2 | 15 | 7742.1 | 7 | 7677.4 | 14 | 6402.1 | 12 | 5751.9 |
| 2011-2014 | 43 | 10278.6 | 23 | 10225.9 | 15 | 8382.4 | 5 | 8719.3 | 12 | 6882.1 | 13 | 5974.6 |
| 2015-2018 | 25 | 10530.2 | 23 | 10386.6 | 11 | 9192.1 | 7 | 9314.7 | 15 | 7043.1 | 11 | 6573.7 |
| 2019-2022 | 21 | 13981.4 | 12 | 14122 | 18 | 12281.1 | 10 | 12236.3 | 12 | 9519.5 | 11 | 9357.7 |

**Table S7: Number of deaths and total person years among older children and adolescents (5-9, 10-14 and 15-19 years) in Magu HDSS by area of residence and 4-year time periods (1995-2022)**

| Time periods | 5-9 | | | | 10-14 | | | | 15-19 | | | |
|---|---|---|---|---|---|---|---|---|---|---|---|---|
| | Semi-urban | | Rural | | Semi-urban | | Rural | | Semi-urban | | Rural | |
| | Deaths | Total person years | Deaths | Total person years | Deaths | Total person years | Deaths | Total person years | Deaths | Total person years | Deaths | Total person years |
| 1995-1998 | 15 | 4943.8 | 29 | 7798.1 | 5 | 3943.8 | 15 | 6333.3 | 5 | 3295.6 | 19 | 4920.9 |
| 1999-2002 | 15 | 5872.1 | 30 | 8366.4 | 4 | 4804.5 | 20 | 6964.0 | 9 | 3657.9 | 18 | 5210.5 |
| 2003-2006 | 15 | 6800.0 | 17 | 9770.1 | 2 | 5871.3 | 14 | 7970.2 | 13 | 4710.8 | 15 | 5838.4 |
| 2007-2010 | 9 | 7457.6 | 31 | 10974.7 | 6 | 6316.6 | 16 | 9102.9 | 7 | 5316.5 | 19 | 6837.5 |
| 2011-2014 | 28 | 8156.7 | 38 | 12347.8 | 7 | 7252.1 | 13 | 9849.6 | 14 | 5648.2 | 11 | 7208.6 |
| 2015-2018 | 15 | 8716.3 | 33 | 12200.5 | 5 | 7855.5 | 13 | 10651.3 | 7 | 6275.4 | 19 | 7341.4 |
| 2019-2022 | 6 | 12161.5 | 27 | 15941.9 | 9 | 10911.3 | 19 | 13606.1 | 11 | 8495.4 | 12 | 10381.7 |

**Table S8: Number of deaths and total person years among older children and adolescents (5-9, 10-14 and 15-19 years) in Magu HDSS by wealth tertiles and 4-year time periods (1995-2022)**

| Time periods | 5-9 | | | | | | 10-14 | | | | | | 15-19 | | | | | |
|---|---|---|---|---|---|---|---|---|---|---|---|---|---|---|---|---|---|---|
| | Poorest 33% | | Middle | | Richest 33% | | Poorest 33% | | Middle | | Richest 33% | | Poorest 33% | | Middle | | Richest 33% | |
| | Deaths | Total person years | Deaths | Total person years | Deaths | Total person years | Deaths | Total person years | Deaths | Total person years | Deaths | Total person years | Deaths | Total person years | Deaths | Total person years | Deaths | Total person years |
| 1995-1998 | 14 | 3931.8 | 14 | 3221.4 | 3 | 2168.3 | 6 | 3281.7 | 3 | 2726.7 | 3 | 1829.8 | 7 | 2490.2 | 5 | 2329.5 | 1 | 1553.6 |
| 1999-2002 | 23 | 5009.1 | 10 | 4001.0 | 6 | 2987.5 | 12 | 4098.3 | 5 | 3474.4 | 4 | 2622.2 | 15 | 2968.0 | 7 | 2687.6 | 2 | 2080.4 |
| 2003-2006 | 12 | 6637.8 | 10 | 5259.7 | 10 | 3980.9 | 7 | 5334.5 | 6 | 4431.4 | 2 | 3584.8 | 9 | 3774.9 | 11 | 3359.6 | 5 | 3057.5 |
| 2007-2010 | 19 | 6796.9 | 12 | 5381.1 | 5 | 4228.3 | 6 | 5822.8 | 10 | 4660.6 | 3 | 3898.7 | 13 | 4273.8 | 7 | 3572.3 | 6 | 3504.5 |
| 2011-2014 | 27 | 7238.2 | 20 | 5785.6 | 15 | 5080.4 | 6 | 5971.5 | 9 | 4975.6 | 4 | 4732.4 | 10 | 4297.0 | 5 | 3681.8 | 8 | 3981.8 |
| 2015-2018 | 21 | 7181.2 | 12 | 6181.4 | 13 | 6397.1 | 7 | 6436.7 | 7 | 5539.6 | 4 | 5818.0 | 11 | 4353.4 | 8 | 4047.1 | 7 | 4744.3 |
| 2019-2022 | 12 | 8478.7 | 14 | 8494.6 | 7 | 10273 | 14 | 7662.1 | 8 | 7450.0 | 5 | 8968.4 | 7 | 5655.2 | 11 | 5744.9 | 5 | 7183.5 |

**Table S9: Average annual rate of change in mortality probabilities trends among older children and adolescents aged 5-19 years compared to children aged 1-4 years in Magu HDSS (1995-2022)**

| | Age groups | | | |
|---|---|---|---|---|
| | **1-4**<br>**AARC[¥] (95% CI)** | **5-9**<br>**AARC[¥] (95% CI)** | **10-14**<br>**AARC[¥] (95% CI)** | **15-19**<br>**AARC[¥] (95% CI)** |
| **Overall** | -4.8 (-5.5, -4.0) *** | -2.0 (-3.3, -0.7) ** | -2.7 (-4.4, -1.1) ** | -2.9 (-4.2, -1.5) *** |
| **Sex** | | | | |
| Male | -5.1 (-5.8, -4.3) *** | -1.7 (-3.0, -0.5) ** | -1.1(-2.7, 0.5) | -2.5 (-3.8, -1.1) *** |
| Female | -4.3 (-5.1, -3.4) *** | -2.6 (-4.0, -1.2) *** | -6.4 (-8.1, -4.7) *** | -2.5 (-3.9, -1.1) *** |
| **Area of residence** | | | | |
| Semi-urban | -5.8 (-6.7, -4.9) *** | -1.2 (-2.7, 0.2) | -1.7 (-3.7, 0.3) | -3.2 (-4.8, -1.6) *** |
| Rural | -4.0 (-4.7, -3.3) *** | -1.8 (-3.0, -0.6) ** | -2.8 (-4.3, -1.3) *** | -3.1 (-4.3, -1.7) *** |
| **Wealth tertiles** | | | | |
| Poorest 33% | -2.3 (-3.0, -1.5) *** | -1.4 (-2.6, -0.1) * | -2.3 (-3.9, -0.8) ** | -3.6 (-4.9, -2.3) *** |
| Middle | -4.6 (-5.4, -3.8) *** | -3.5 (-4.7, -2.2) *** | -1.3 (-3.1, 0.4) | -1.7 (-3.2, -0.3) * |
| Richest 33% | -6.6 (-7.5, -5.7) *** | -0.2 (-1.9, 0.8) | -5.7 (-7.4, -3.9) *** | -4.1 (-5.8, -2.3) ** |

*[¥]Average annual rate of change; *p<0.05, ** p<0.01, ***p<0.001*

## Comparison with global estimates

**Table S10: Four-year averaged mortality probabilities estimate, comparing population data estimates from Magu HDSS with global estimates (UN IGME, GBD)**

| Time period | Magu HDSS estimate | UN IGME point estimate | GBD point estimate | Relative difference UN IGME (%) * | Relative difference GBD (%) ¥ |
|---|---|---|---|---|---|
| | | | ***1–4-year-olds*** | | |
| **1990-1994** | | 72.3 | 166.3 | | |
| **1995-1998** | 63.6 | 66.7 | 149.8 | -4.8 | -135.5 |
| **1999-2002** | 54.3 | 53.6 | 127.0 | 1.3 | -133.8 |
| **2003-2006** | 36.2 | 39.2 | 101.1 | -8.4 | -179.2 |
| **2007-2010** | 42.7 | 29.1 | 82.2 | 31.9 | -92.6 |
| **2011-2014** | 40.0 | 21.2 | 66.2 | 46.9 | -65.4 |
| **2015-2018** | 18.7 | 16.6 | 52.0 | 11.1 | -177.8 |
| | | | ***5–9-year-olds*** | | |
| **1990-1994** | | 18.7 | 15.9 | | |
| **1995-1998** | 16.9 | 16.4 | 16.0 | 3.2 | 5.5 |
| **1999-2002** | 15.6 | 13.7 | 14.7 | 12.1 | 5.8 |
| **2003-2006** | 9.6 | 11.6 | 12.8 | -20.4 | -33.8 |
| **2007-2010** | 10.7 | 10.3 | 11.8 | 3.8 | -10.0 |
| **2011-2014** | 15.7 | 10.1 | 10.2 | 35.8 | 35.2 |
| **2015-2018** | 11.4 | 9.9 | 9.0 | 13.5 | 21.2 |
| | | | ***10–14-year-olds*** | | |
| **1990-1994** | | 7.9 | 9.9 | | |
| **1995-1998** | 9.9 | 6.8 | 9.7 | 31.7 | 1.9 |
| **1999-2002** | 10.5 | 5.6 | 9.8 | 46.8 | 6.5 |
| **2003-2006** | 5.7 | 4.7 | 9.8 | 18.0 | -71.1 |
| **2007-2010** | 7.3 | 4.1 | 9.5 | 43.5 | -30.6 |
| **2011-2014** | 5.9 | 4.0 | 8.1 | 32.2 | -36.7 |
| **2015-2018** | 5.0 | 3.9 | 7.2 | 21.5 | -43.9 |
| | | | ***15–19-year-olds*** | | |
| **1990-1994** | | 14.2 | 19.5 | | |
| **1995-1998** | 14.3 | 15.5 | 18.0 | -8.7 | -25.8 |
| **1999-2002** | 14.8 | 13.9 | 16.6 | 6.0 | -11.9 |
| **2003-2006** | 12.9 | 10.8 | 16.7 | 16.0 | -29.8 |
| **2007-2010** | 10.9 | 8.0 | 16.5 | 26.8 | -51.3 |
| **2011-2014** | 9.8 | 7.7 | 14.6 | 21.0 | -49.1 |
| **2015-2018** | 9.9 | 8.0 | 12.4 | 19.6 | -25.1 |
